# Supplementary material for: Leveraging Gene Redundancy to Find New Histone Drivers in Cancer
Source: Cancers (Basel). 2023 Jun 30;15(13):3437. doi: 10.3390/cancers15133437 (PMC10340511; doi:10.3390/cancers15133437)

Supplementary Information
Leveraging Gene Redundancy to Find New Histone Drivers in Cancer

Daria Ostroverkhova, Daniel Espiritu, Maria J. Aristizabal and Anna R. Panchenko

Supplementary Figure S1. A volcano plot showing the statistical significance (q-values) versus the fold change in mean mutation rate for histone versus known CGC driver non-histone proteins in various cancer types. Fold change has been calculated as (*mean histone mutation rate*) / (*mean non-histone mutation rate*). q-values have been plotted on the y-axis on a $-\log_{10}$ scale (Mann-Whitney U, Benjamini Hochberg, FDR = 0.05). The green line corresponds to a q = 0.05. Labels have been provided for cancer types with a significant difference between the mutation rates of histone proteins and non-histone proteins ($q < 0.05$). Furthermore, points where Fold Change > 1 and $q < 0.05$ have been colored blue. Points where Fold Change < 1 and $q < 0.05$ have been colored red.

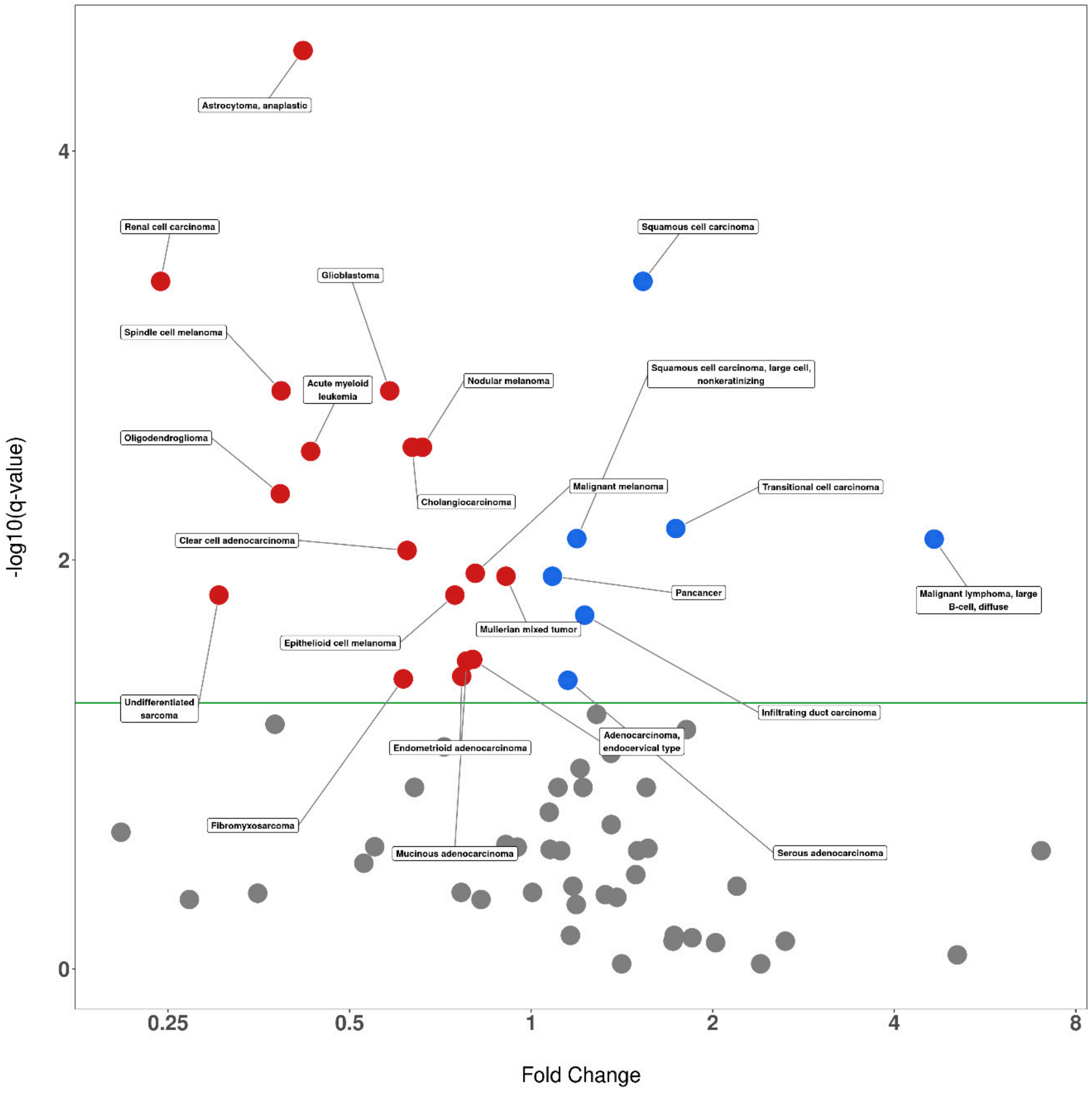

Supplementary Figure S2. A: Replication Time plotted for chromosome location of each gene. **B:** Hi-C levels plotted versus replication time, **C:** Hi-C levels plotted versus expression level, **D:** Expression level plotted versus replication time. Variants are shown in blue dots, canonical genes are shown in red dots. Predicted histone driver genes are labelled. Several points corresponding to canonical genes overlap with each other since they have the same replication time.

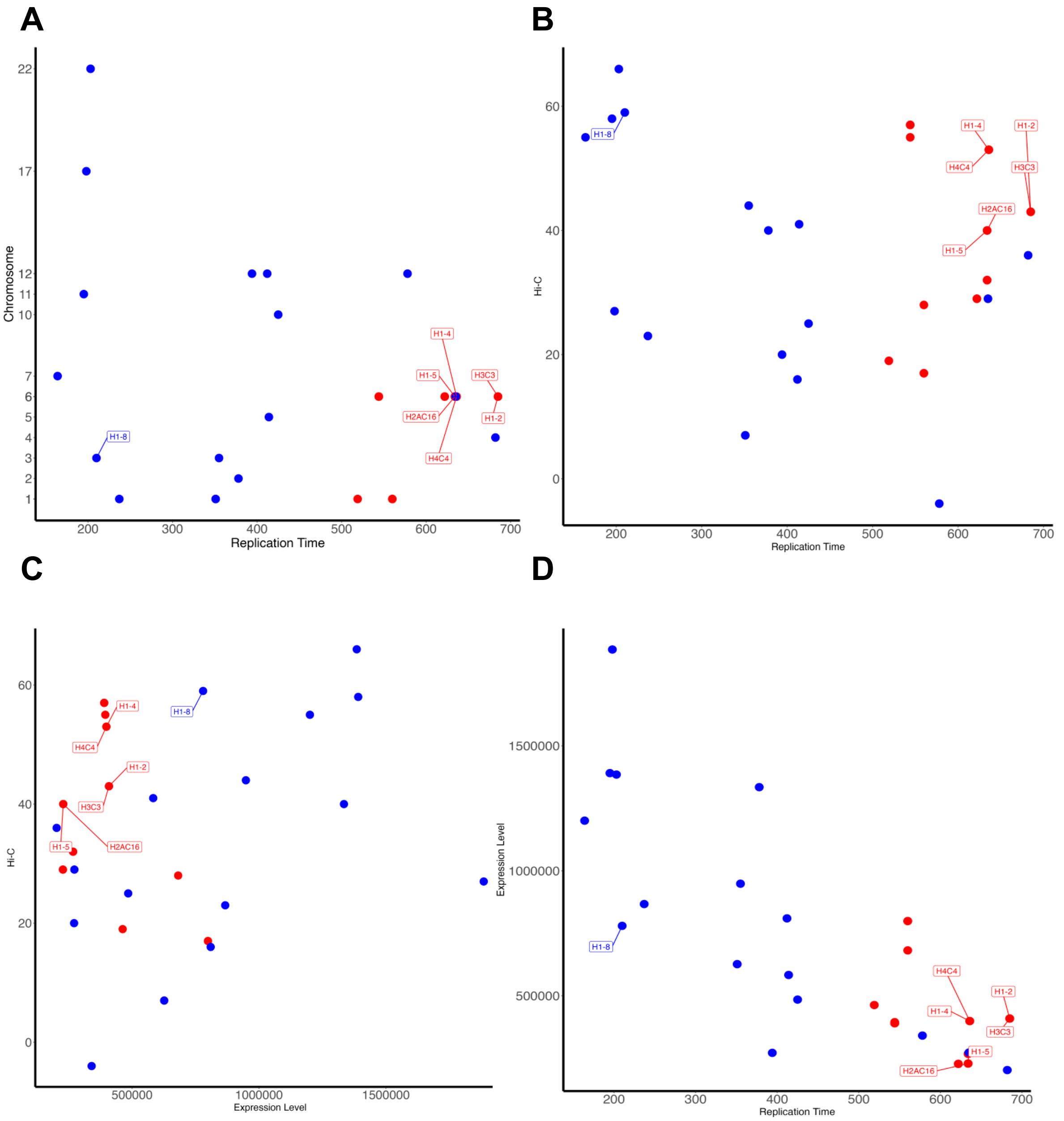

Supplementary Figure S3. Mutational profiles of three gene cohorts used in the study. A: Mutational profiles of mutated histone genes (gene_group1), **B:** mutated non-histone genes (gene_group2) and **C:** mutated genes from patients which do not harbor histone mutations (gene_group3).

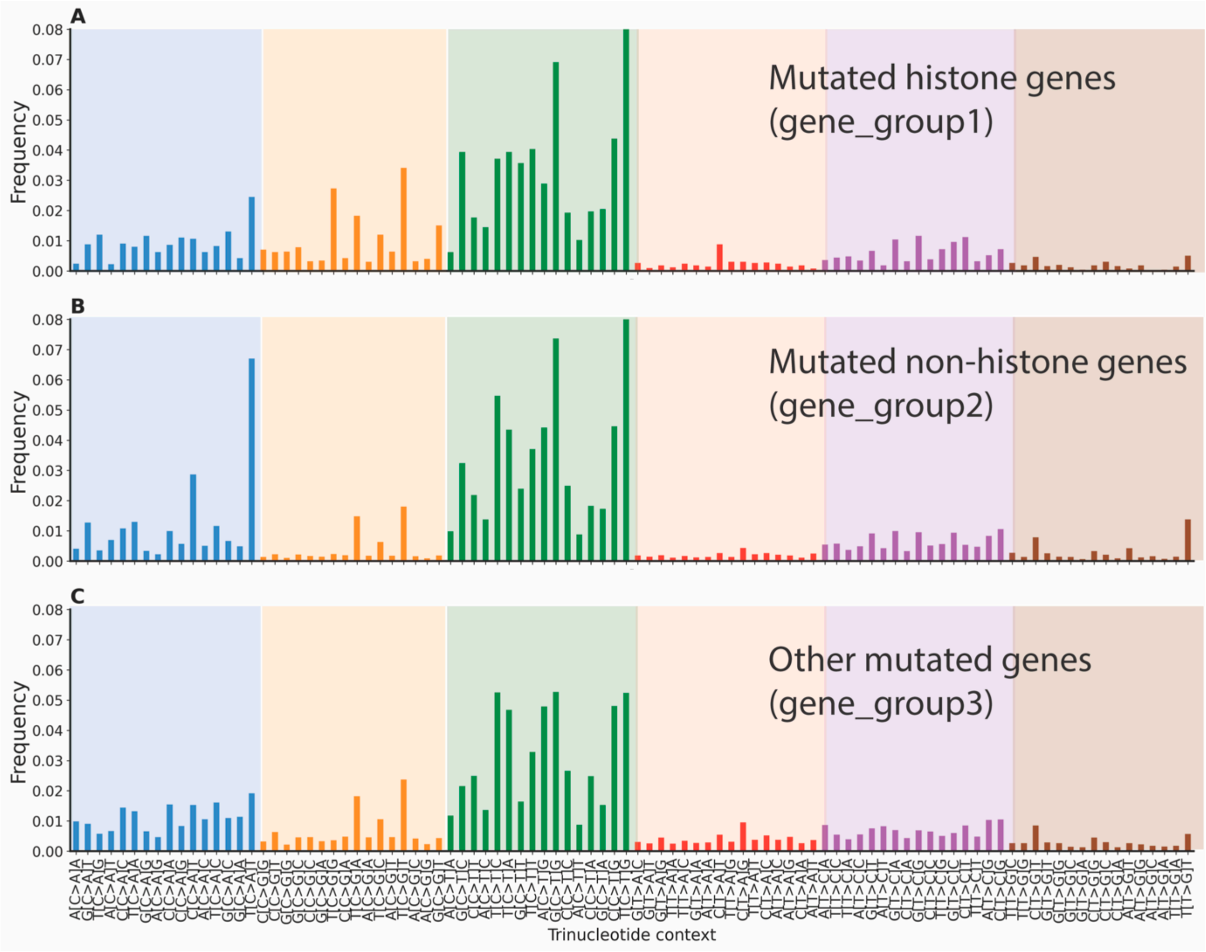

Supplement: Supplementary file 1 [file cancers-15-03437-s001.zip › cancers-2446076-Figures.pdf]
